# The role of regulatory T cells in the pathogenesis of acute kidney injury

**DOI:** 10.1111/jcmm.17771

**Published:** 2023-09-04

**Authors:** Xiaoyou Liu, Jianmin Hu, Guorong Liao, Ding Liu, Song Zhou, Jie Zhang, Jun Liao, Zefeng Guo, Yuzhu Li, Siqiang Yang, Shichao Li, Hua Chen, Ying Guo, Min Li, Lipei Fan, Liuyang Li, Ming Zhao, Yongguang Liu

**Affiliations:** ^1^ Department of Organ transplantation The First Affiliated Hospital of Guangzhou Medical University Guangzhou China; ^2^ Department of Organ transplantation Zhujiang Hospital of the Southern Medical University Guangzhou China

**Keywords:** acute kidney injury, immune microenvironment, inflammatory, Tregs

## Abstract

The incidence of acute kidney injury (AKI) is on the rise and is associated with high mortality; however, there are currently few effective treatments. Moreover, the relationship between Tregs and other components of the immune microenvironment (IME) in the pathogenesis of AKI remains unclear. We downloaded four publicly accessible AKI datasets, GSE61739, GSE67401, GSE19130, GSE81741, GSE19288 and GSE106993 from the gene expression omnibus (GEO) database. Additionally, we gathered two kidney single‐cell sequencing (scRNA‐seq) samples from the Department of Organ Transplantation at Zhujiang Hospital of Southern Medical University to investigate chronic kidney transplant rejection (CKTR). Moreover, we also collected three samples of normal kidney tissue from GSE131685. By analysing the differences in immune cells between the AKI and Non‐AKI groups, we discovered that the Non‐AKI group contained a significantly greater number of Tregs than the AKI group. Additionally, the activation of signalling pathways, such as inflammatory molecules secretion, immune response, glycolytic metabolism, NOTCH, FGF, NF‐κB and TLR4, was significantly greater in the AKI group than in the Non‐AKI group. Additionally, analysis of single‐cell sequencing data revealed that Tregs in patients with chronic kidney rejection and in normal kidney tissue have distinct biology, including immune activation, cytokine production, and activation fractions of signalling pathways such as NOTCH and TLR4. In this study, we found significant differences in the IME between AKI and Non‐AKI, including differences in Tregs cells and activation levels of biologically significant signalling pathways. Tregs were associated with lower activity of signalling pathways such as inflammatory response, inflammatory molecule secretion, immune activation, glycolysis.

## 1 INTRODUCTION

Acute kidney injury (AKI) is a clinical syndrome in which renal impairment triggers the accumulation of nephrotoxins, which in turn leads to impaired function of multiple organs.^1^ In ICU patients, the mortality rate for patients with AKI is 1.5–2 times higher than that of patients without AKI, indicating that AKI can be an independent risk factor for death in the ICU.^2^ The incidence of AKI is on the rise, and AKI has become a major global health issue associated with high mortality.^3^ According to studies, the pathogenesis of AKI is complex, and despite extensive research, the pathogenesis of AKI is still not fully understood, and there are no effective clinical treatments.^4^ The pathogenesis of AKI varies significantly depending on the aetiology, and reports on AKI pathogenesis include inflammation, ischemia–reperfusion, and renal tubular injury.^5^, ^6^

Numerous studies have demonstrated that the immune microenvironment (IME) plays a crucial role in AKI.^7^, ^8^ The immune response network is intricate, and different immune microenvironmental components have been found to play distinct roles in AKI.^9^, ^10^, ^11^ Resident immune cells and intrinsic renal cells are damaged by ischemia, hypoxia, drugs, and toxins and recruit more immune cell infiltration by releasing chemokines, while intrinsic cells promote macrophage polarity switching and immune cells promote various programmed deaths, intrinsic cell phenotypic switching, and cycle arrest, resulting in renal impairment.^7^

Regulatory T cells (Tregs), which express high levels of CD25 and the transcription factor forkhead box protein 3 (Foxp3), are essential for immune homeostasis.^12^ Several animal studies have demonstrated that Tregs play a renoprotective role in models of renal ischemia–reperfusion and drug‐induced renal injury.^13^ However, the association between Tregs and other immune microenvironmental components (e.g., immune cells or signalling pathways) in the pathogenesis of AKI remains unclear.

In this study, we analysed the IME in AKI using publicly available transcriptomic data from four AKI projects. Additionally, we compared the immune microenvironments of AKI and non‐AKI samples. We also collected scRNA‐seq samples from the Department of Organ Transplantation at Southern Medical University's Zhujiang Hospital. Specifically, we collected two scRNA‐seq samples of chronic kidney transplant rejection (CKTR) and three scRNA‐seq samples of normal kidney tissues from publicly available databases to investigate the functional heterogeneity of Treg.

## 2 METHODS

### 2.1 AKI cohort

**FIGURE 1:**
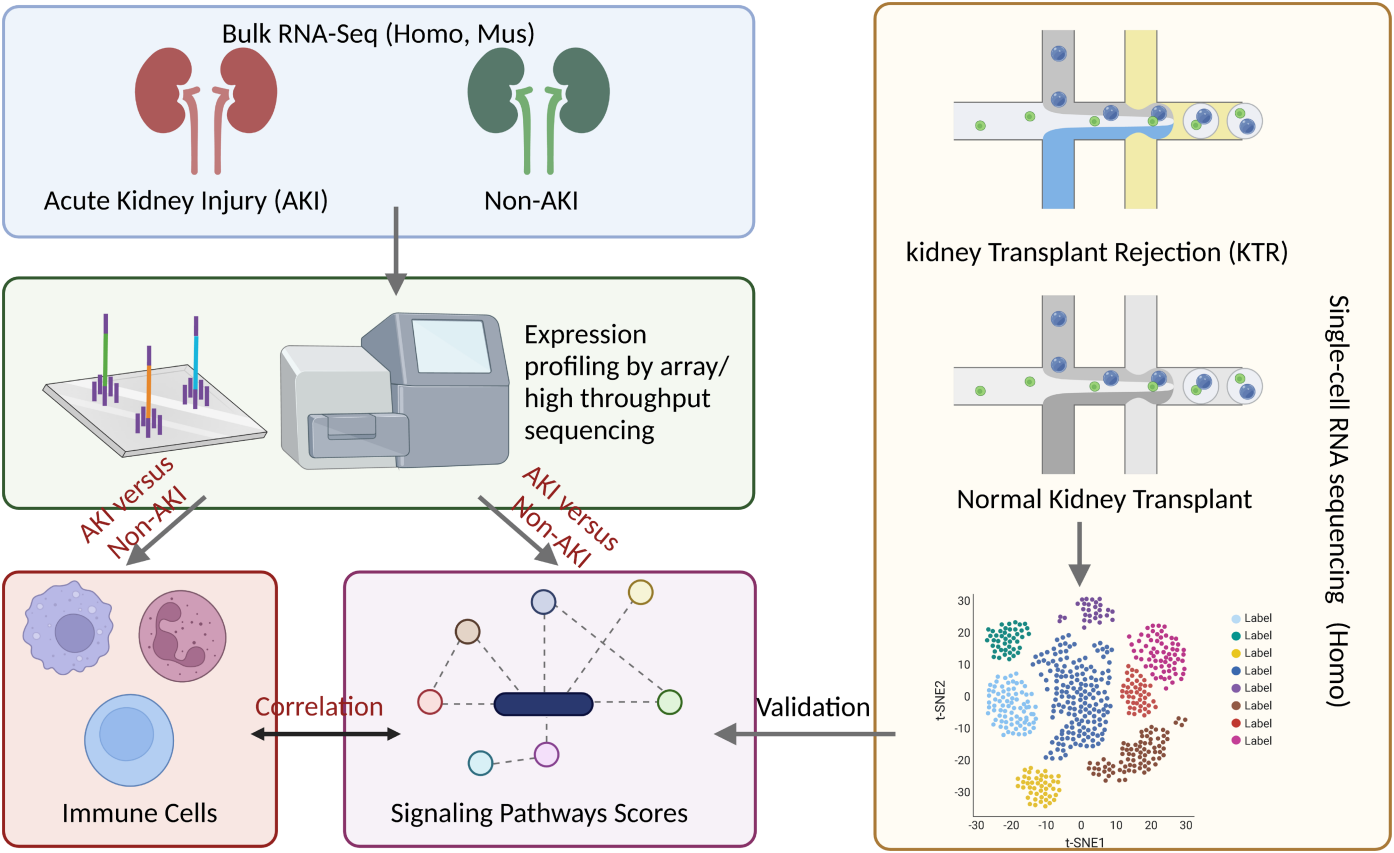
The overall design of the study.

In GEO database, we searched and downloaded four publicly available AKI datasets from the GEO database: GSE61739^14^ (Homo, 48 AKI versus 48 Non‐AKI), GSE67401^15^ (Homo, 111 AKI versus 22 Non‐AKI), GSE19130^16^ (Homo, 28 AKI versus 48 Non‐AKI), GSE81741^17^ (Mus, 36 AKI versus 14 Non‐AKI), GSE192883^18^ (Mus, 24 AKI versus 3 Non‐AKI) and GSE106993^19^ (Mus, 20 AKI versus 20 Non‐AKI). All four AKI datasets contain transcriptomic and clinical data on AKI (Figure 1). The transformation between gene symbol and ID in GSE61739 and GSE19130 (Homo) was performed using GPL13158 and GPL9301, respectively. Each AKI cohort includes both AKI and Non‐AKI samples. Non‐AKI group contained non‐rejection or renal recipients with stable function.

### 2.2 Single‐cell sequencing analysis

We collected scRNA‐seq samples from two cases of CKTR from the Department of Organ Transplantation of Zhujiang Hospital of Southern Medical University.^20^ The Ethics Committee of Zhujiang Hospital of Southern Medical University reviewed all patient samples and issued ethical approval consent. The samples from CKTR were sequenced using scRNA‐seq methods (Supplementary Methods). We also downloaded scRNA‐seq data (GSE131685)^21^ for three normal kidneys from the GEO database.

### 2.3 Immune cell fraction assessment

We utilized the QUANTISEQ,^22^ EPIC,^23^ xCell^24^ and ssGSEA algorithms^20^ to evaluate the immune cell fraction of each AKI sample using the expression data from the AKI dataset. The quantiseq algorithm can, for instance, evaluate B cells, M1 Macrophages, M2 Macrophages, Monocytes, NK cells, CD4 T cells, CD8 T cells, Tregs, and Dendritic cells. B cells, CD4 T cells, CD8 T cells, Endothelial, Macrophages, and NK cells can all be evaluated by the ssGSEA algorithm.^25^

### 2.4 Pathway analysis

We compared the degree of difference between AKI and non‐AKI samples' transcriptome gene expression. Log2 fold change (logFC) and ENTREZID of AKI and Non‐AKI on the transcriptome were then used as input files for the GSEA algorithm.^26^ Subsequently, using the pathway collections from the GO‐BP, GO‐CC, GO‐MF, KEGG, and Reactome databases, we compared the levels of pathway activation in AKI and Non‐AKI samples (mainly in terms of enrichment scores and p values). In addition, we utilized the ssGSEA algorithm and the MsigDB pathway collection to calculate the ssGSEA scores for each sample's pathway.

### 2.5 Statistical methods

The U‐test was used to compare AKI and Non‐AKI samples for differences in continuous variables. Next, heatmaps were produced using the ‘ComplexHeatmap’ R package,^27^ and scatter plots were produced using the ‘ggstatsplot’ R package. The ‘org.Mm.eg.db’ R package was used for transformation between mouse ENTREZID and gene symbol. Probability values less than 0.05 were deemed statistically significant (*p*‐values are bilateral). Previous literature was consulted for information on the data analysis and visualization process following single‐cell sequencing.^20^

## 3 RESULTS

### 3.1 Relationship between AKI and Tregs

**FIGURE 2:**
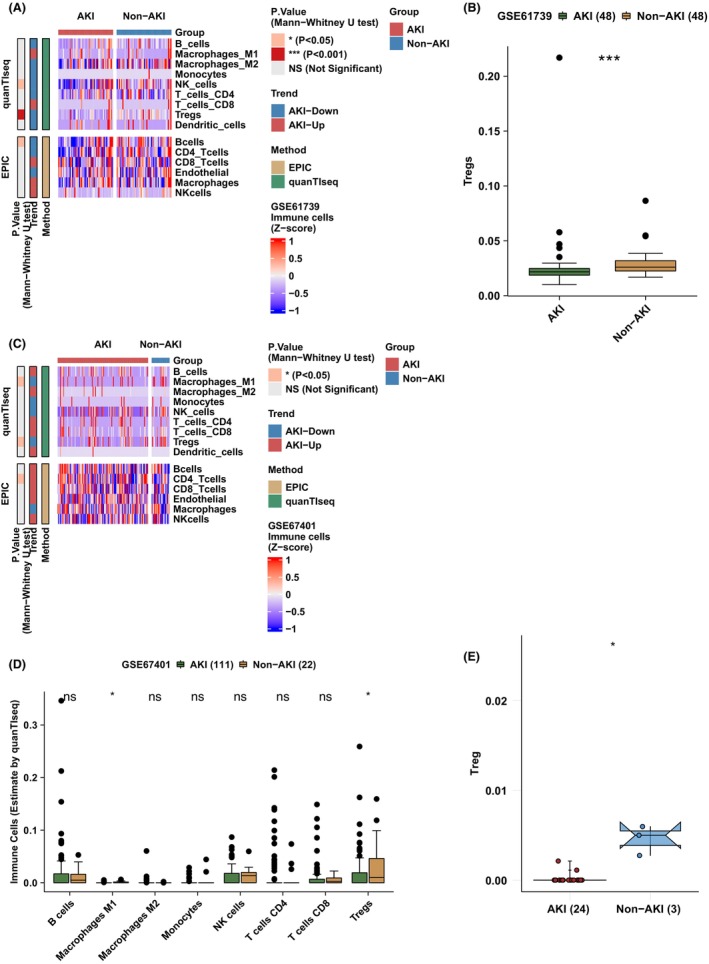
The differences between the AKI and non‐AKI groups with regard to Tregs. Heatmap (A) and boxplot (B) illustrated the differences in Tregs between the AKI and Non‐AKI group in GSE61739. Heatmap (C) and boxplot (D) illustrated the differences in Tregs between the AKI and Non‐AKI group in GSE67401. (E) The boxplot illustrated the differences in Tregs between the AKI and Non‐AKI groups in GSE192883.

We compared AKI and Non‐AKI groups in the immune microenvironment (Figure 1) to investigate the differences between AKI and Non‐AKI groups in Tregs (Figure 2). In GSE61739, we discovered that the Non‐AKI group had significantly more Tregs than the AKI group (Figure 2A,B, *p* < 0.05). Similarly, the Non‐AKI group in GSE67401 had significantly higher Tregs than the AKI group (Figure 2C,D, *p* < 0.05). Additionally, we discovered that Non‐AKI had significantly higher Treg scores than AKI in the mouse model (GSE192883: Figure 2E, *p* < 0.05). In terms of immune cells, we also analysed the association between the AKI and Non‐AKI groups in the GSE61739 and GSE67401 datasets (Figure S1A,B). In summary, we found that the Non‐AKI group had significantly increased Treg cells compared with the AKI group.

### 3.2 Differences in the degree of signalling pathway activation between AKI and Non‐AKI

**FIGURE 3:**
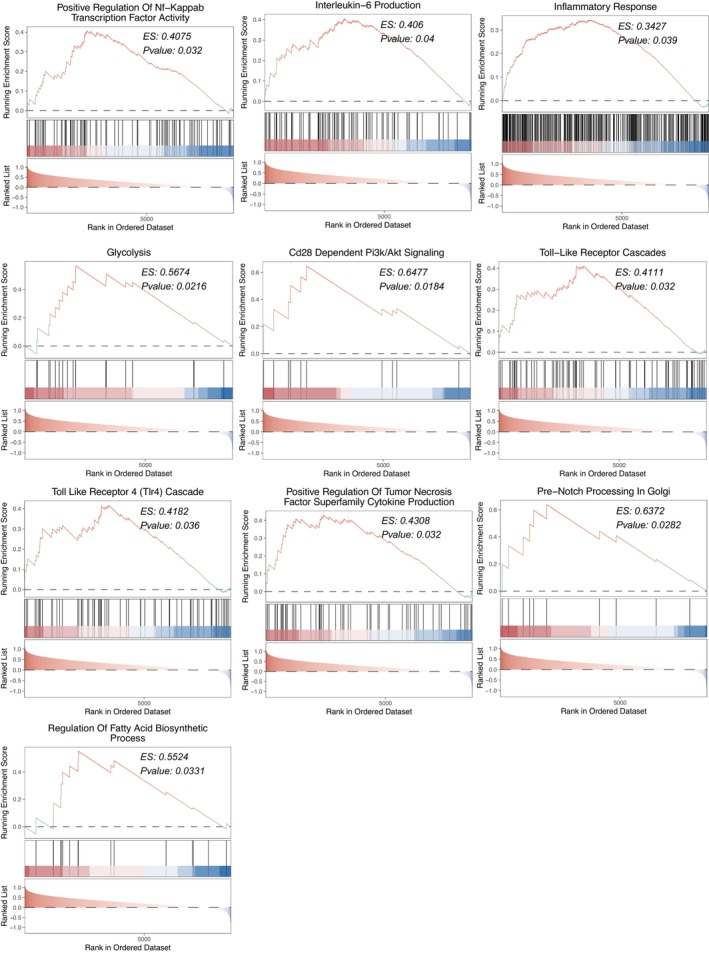
The gene set enrichment analysis (GSEA) of the AKI group compared with the Non‐AKI group.

To compare the differences in signalling pathway scores between patients in AKI and Non‐AKI groups, we performed pathway enrichment analysis of transcriptomic data from AKI and Non‐AKI patients. Inflammatory signalling pathways [such as regulation of fatty acid biosynthetic process, positive regulation of tumour necrosis factor superfamily cytokine production, positive regulation of NF‐kappaB transcription factor activity, interleukin‐6 production, inflammatory response, CD28 dependent PI3K/Akt signalling, Pre‐NOTCH Processing in Golgi, Glycolysis, Toll Like Receptor 4 (TLR4) Cascade, Toll‐like Receptor Cascades] were significantly activated in the AKI group, compared to Non‐AKI (Figure 3). In summary, we found that the AKI group had significantly increased the activity of the inflammatory signalling pathways compared with the Non‐AKI group.

### 3.3 The relationship between Treg and signalling pathways

**FIGURE 4:**
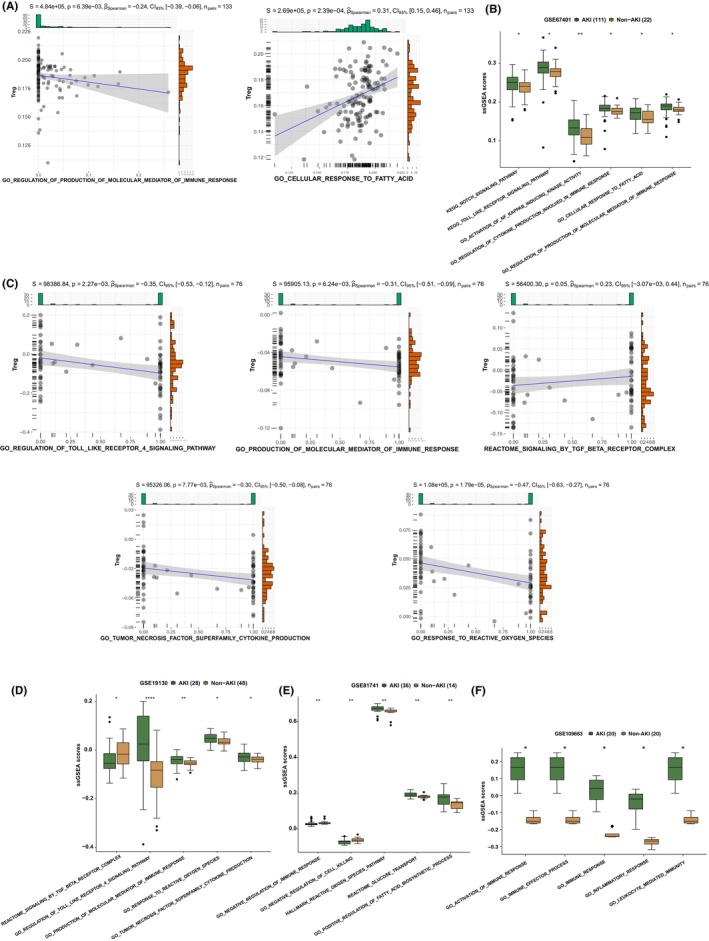
The activity of signalling pathways in AKI and the correlation between Tregs and signalling pathway scores. (A) Correlation between the signalling pathway scores estimated by ssGSEA and the Tregs in GSE67401. (B) The differences in signalling pathway scores between the AKI and Non‐AKI groups in the GSE67401, as estimated by ssGSEA. (C) The correlation between the signalling pathway scores determined by ssGSEA and the Tregs in the GSE19130. The differences in signalling pathway scores were estimated by ssGSEA between the AKI and Non‐AKI groups in the GSE19130 (D), GSE81741 (E) and GSE106993 (F).

In GSE67401, we found a significant positive correlation between Tregs and GO cellular response to fatty acid in GSE67401. A significant negative correlation was observed between Tregs and GO regulation of the production of molecular mediators of immune response (Figure 4A). Additionally, we discovered that the AKI group had significantly higher signal activation than the Non‐AKI group on the following pathways: KEGG NOTCH SIGNALLING PATHWAY, KEGG TOLL LIKE RECEPTOR SIGNALLING PATHWAY, GO ACTIVATION OF NF KAPPAB INDUCING KINASE ACTIVITY, GO REGULATION_OF_CYTOKINE_PRODUCTION_INVOLVED_IN_IMMUNE_RESPONSE, GO_CELLULAR _RESPONSE_TO_FATTY_ACID, GO_REGULATION_OF_PRODUCTION_OF_MOLECULAR _MEDIATOR _OF_IMMUNE_RESPONSE score (Figure 4B, GSE61739). Furthermore, in GSE19130, we found a significant negative correlation between Treg cell fraction and REACTOME SPRY REGULATION OF FGF SIGNALLING, REACTOME SIGNALLING BY NOTCH3, REACTOME GLYCOLYSIS, REACTOME FATTY ACIDS, GO REGULATION OF FIBROBLAST GROWTH FACTOR _RECEPTOR_SIGNALLING_PATHWAY, GO_POSITIVE_REGULATION_OF_TRANSFORMING_GROWTH_FACTOR_BETA_PRODUCTION, GO_TYPE_I_INTERFERON_RECEPTOR_BINDING signalling pathway fraction (Figure 4C). In addition, we discovered that the AKI group had significantly higher signalling activation scores on the aforementioned pathway than the Non‐AKI group (Figure 4D, GSE19130). In GSE81741 (mice model), we discovered that the AKI group had significantly higher GO NEGATIVE REGULATION OF IMMUNE RESPONSE and GO NEGATIVE REGULATION OF CELL KILLING signalling pathway scores than the Non‐AKI group (Figure 4E). In contrast, the AKI group had significantly higher pathway scores for HALLMARK_REACTIVE_OXIGEN_SPECIES_PATHWAY, REACTOME_GLUCOSE_TRANSPORT, GO_POSITIVE_ REGULATION_OF_FATTY_ACIDBIOSYNTHETIC_PROCESS pathway scores than the Non‐AKI group (Figure 4E). Also, we found that AKI mice had significantly increased GO_ACTIVATION_OF_IMMUNE_RESPONSE, GO_IMMUNE_EFFECTOR_PROCESS, GO_IMMUNE_RESPONSE, GO_INFLAMMATORY_RESPONSE, and GO_LEUKOCYTE_MEDIATED_IMMUNITY pathway scores than the Non‐AKI group (Figure 4F: GSE106993). In summary, we found that there was a negative correlation between the proportion of the Tregs cells and immune response‐related signalling pathways.

### 3.4 Different functions in different subtypes of Tregs

**FIGURE 5:**
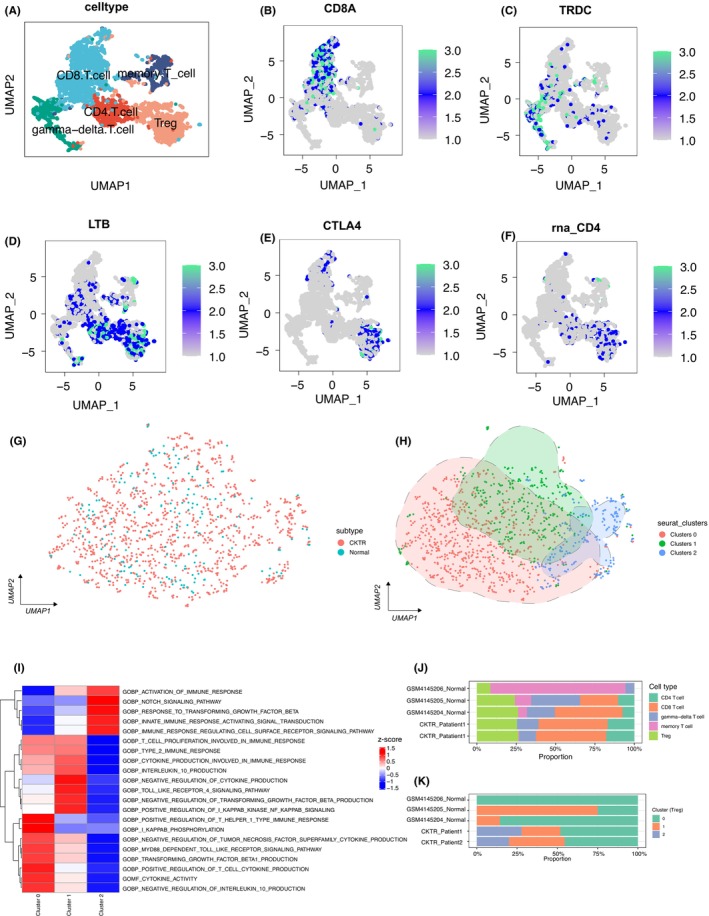
The biological function of Tregs as determined by single‐cell sequencing. (A) The UMAP plot displayed T cell clusters. (B) UMAP plot colour‐coded for the expression of the marker genes for CD4+ T cells (A), CD8+ T cells (B), gamma delta T cells (C), memory T cells (D), and Tregs (E, F). (G) Tregs' sample origin is colour‐coded on their UMAP plot. (H) The UMAP plot illustrated the Tregs clusters. (I) Heatmap of the ssGSEA score for three Tregs clusters, as estimated using gene sets from MsiDB. (J) The proportion of the cell types of each sample. (K) The differences in the Tregs subtypes between the normal and CKTR group.

According to previously published literature, we clustered five single‐cell sequencing samples to define each cluster of cells (Figure S2A). We found that the marker gene expression in each cluster of cells further validated the classification's reliability (Figure S2B, Table S1). Treg cells were isolated and clustered to further investigate the functional heterogeneity of Tregs (Figure 5A). CD8+ T cells (Figure 5B), gamma delta T cells (Figure 5C), memory T cells (Figure 5D), Tregs (Figure 5E), and CD4+ T cells were identified as five distinct T cell clusters (Figure 5F). Figure 5G depicts the cellular descending distribution of Treg cells in the chronic kidney rejection group and normal kidney tissue. Tregs were then clustered and divided into three clusters, Cluster 0, Cluster 1, and Cluster 2 (Figure S3, Figure 5H). We used the ssGSEA algorithm to perform pathway enrichment analysis for each cluster (Figure 5I), and we discovered that each Tregs subpopulation has distinct biologically relevant functions, e.g., Cluster 2 has significantly upregulated immune response‐related and NOTCH pathways and significantly downregulated cytokine‐related, NF‐κB and TLR4 signalling pathways. Figure 5J showed the proportion of the cell types of each sample, we found that there was a certain proportion of Tregs among all the samples. Next, we compared the differences in the Tregs subtypes between the normal and CKTR group. We found that the Cluster 2 Tregs were enriched in the CKTR group compared with the normal group (Figure 5K, Table S2). These results indicated that the biological function of Treg cells in CKTR patients and normal kidney tissue may differ.

## 4 DISCUSSION

In this study, we discovered that the AKI group contained significantly fewer Tregs than the Non‐AKI group. Additionally, the activation of signalling pathways, including inflammatory factor secretion, immune response, glycolytic metabolism, NOTCH, FGF, NF‐κB, and TLR4, was significantly greater in the AKI group than in the Non‐AKI group. In addition, correlation analyses indicated that Treg was significantly associated with lower activation scores of inflammatory factor secretion, immune response, glycolytic metabolism, NOTCH, FGF, NF‐κB, and TLR4 signalling pathways. We found that the Cluster 2 Treg cells were enriched in the CKTR group compared with the normal group. Cluster 2 has significantly upregulated immune response‐related and NOTCH pathways and significantly downregulated cytokine‐related, NF‐κB and TLR4 signalling pathways. Moreover, the analysis of ScRNA‐seq data revealed that the biological function of Treg cells in CKTR patients and normal kidney tissue may differ.

The IME with a lower ratio of Tregs may be one of the key mechanisms of AKI, a CD4+ T cell subpopulation with potent immunosuppressive effects and regulation of peripheral immune tolerance.^12^, ^28^ In animal models, targeting Tregs with a monoclonal antibody against CD25 can exacerbate the inflammatory response in the kidney, resulting in tubular necrosis and impaired renal function.^29^ Tregs induce adenosine production by releasing IL‐10 in the sphingosine‐induced ischemia/reperfusion (I/R) model, which encodes PD‐1 on the cell surface,^30^ inhibits inflammatory and immune responses, protects kidney cells, and promotes repair.^31^ Kinsey et al.^29^ discovered that the transfer of lymph nodes from wild‐type mice and Foxp3 knockout mice to T‐ and B‐cell‐deficient mice caused the production of FoxP3‐positive T cells in the wild‐type mice. Similarly, Lai et al.^32^ found that after ischemia–reperfusion kidney injury in mice, sphingosine kinase inhibitor application increased the number of Tregs that migrated to the injury and protected the kidney.

The IME has a markedly increased inflammatory response, and inflammatory factor secretion and immune activation pathway activity may be one of the key mechanisms underlying AKI. NF‐κB is a key transcription factor for several inflammatory factors and induces the synthesis and release of TNF‐α, IL‐1, IL‐6, and IL‐8.^33^ Animal experiments have demonstrated that direct inhibition of NF‐κB can reduce inflammatory factor levels.^34^ NF‐κB also induces the transcription of multiple inflammatory factors, which initiates an inflammatory cascade and creates an inflammatory environment in the kidney. Mar et al.^35^ discovered that the epigenetic marker gene (TNF) transcription was activated in an animal model of AKI induced by ischemia–reperfusion, indicating that epigenetic involvement in the process of AKI not only promotes the inflammatory response and activates fibrogenic cytokines but also upregulates the expression of chemokines.^36^ In this study, we discovered that the AKI group not only had a significantly higher inflammatory response, inflammatory factor secretion, and immune activation pathway activity than the Non‐AKI group but that Treg was significantly associated with lower inflammatory response, inflammatory factor secretion, and immune activation pathway activity. Although renal tubular epithelial cells (RTECs) derive little energy from glycolysis under physiological conditions, it was discovered that during the initial phase of acute kidney injury (AKI), upregulated hypoxia‐inducible factors,^37^, ^38^ among others, can activate the glycolytic pathway in RTECs by upregulating key glycolytic pathway enzymes such as membrane glucose transporter protein‐1 (Glut‐1)^39^ and pyruvate kinase M2 (M2‐PK).^40^ NOTCH signalling is a highly conserved intercellular communication mechanism that regulates cell development, tissue homeostasis, and tissue repair.^41^ Kavvadas et al.^42^ discovered an abnormally high level of NOTCH3 expression in a mouse model of ischemia–reperfusion kidney injury. They confirmed that NOTCH3 is involved in the renal inflammatory response and causes tubular epithelial cell injury. NOTCH3 targeting could offer a novel therapeutic approach for acute kidney injury. Targeting NOTCH3 could offer a novel therapeutic approach for acute kidney injury. Moreover, regarding the TLR4/ NF‐κB signalling pathway, during sepsis, LPS enters the renal tubule via the site of infection or distal injury via renal tubular filtration and other mechanisms, resulting in widespread expression of TLR4 in the kidney, which recognizes LPS and activates NF‐κB. In the meantime, it has been demonstrated that NF‐κB can induce the transcription of multiple inflammatory factors, resulting in an inflammatory cascade response and a renal inflammatory environment.^43^ In this study, we not only found that the AKI group had significantly increased glycolysis, NOTCH, and TLR4‐related signalling pathway activity than the Non‐AKI group, but we also discovered that Treg was significantly associated with lower glycolysis, NOTCH, and TLR4‐related signalling pathway activity. There were some limitations in the study. The basic clinical information of publicly accessible AKI datasets GSE61739, GSE67401, GSE19130, and GSE81741 did not contain age, gender, or other clinical characteristics. Thus, we were not able to explore the relationship between Treg cells difference with disease clinical information.

## 5 CONCLUSIONS

The present study compared the IME of AKI and Non‐AKI and revealed that AKI had a significantly lower proportion of Tregs than Non‐AKI. Additionally, Tregs were associated with decreased activity of signalling pathways such as inflammatory response, inflammatory factor secretion, immune activation, glycolysis, and signalling pathways related to pathology. The AKI group had greater activation of the aforementioned signalling pathways than the Non‐AKI group. Consequently, Tregs may play a crucial role in the pathogenesis of AKI.

## AUTHOR CONTRIBUTIONS

**Xiaoyou Liu:** Formal analysis (equal); visualization (equal); writing – review and editing (equal). **Jianmin Hu:** Formal analysis (equal); writing – original draft (equal). **Guorong Liao:** Formal analysis (equal); writing – original draft (equal). **Ding Liu:** Formal analysis (equal); writing – review and editing (equal). **Song Zhou:** Formal analysis (equal); writing – review and editing (equal). **Jie Zhang:** Formal analysis (equal); writing – review and editing (equal). **Jun Liao:** Formal analysis (equal); writing – original draft (equal). **Zefeng Guo:** Formal analysis (equal); writing – review and editing (equal). **Yuzhu Li:** Formal analysis (equal); writing – review and editing (equal). **Siqiang Yang:** Formal analysis (equal); writing – review and editing (equal). **Shichao Li:** Writing – review and editing (equal). **Hua Chen:** Writing – original draft (equal). **Ying Guo:** Writing – original draft (equal). **Min Li:** Writing – original draft (equal). **Lipei Fan:** Writing – original draft (equal). **Liuyang Li:** Writing – original draft (equal). **Ming Zhao:** Conceptualization (equal); formal analysis (equal); writing – review and editing (equal). **Yongguang Liu:** Conceptualization (equal); formal analysis (equal); writing – review and editing (equal).

## FUNDING INFORMATION

This work was supported by Basic and Applied Basic Research Foundation of Guangdong Province (Grant No. 2022A1515012304), and the National Natural Science Foundation of China (Grant No. 82170764).

## CONFLICT OF INTEREST STATEMENT

The authors declare that the research was conducted in the absence of any commercial or financial relationships that could be construed as a potential conflict of interest.

## Supporting information


Figure S1

Figure S2

Figure S3

Data S1

Table S1

Table S2

## Data Availability

All the data generated or analysed during this study are included in this article and its Supporting Information.
