# Supplementary figures and images for: The role of regulatory T cells in the pathogenesis of acute kidney injury
Source: J Cell Mol Med. 2023 Sep 4;27(20):3202–12. doi: 10.1111/jcmm.17771 (PMC10568672; doi:10.1111/jcmm.17771)

**A**

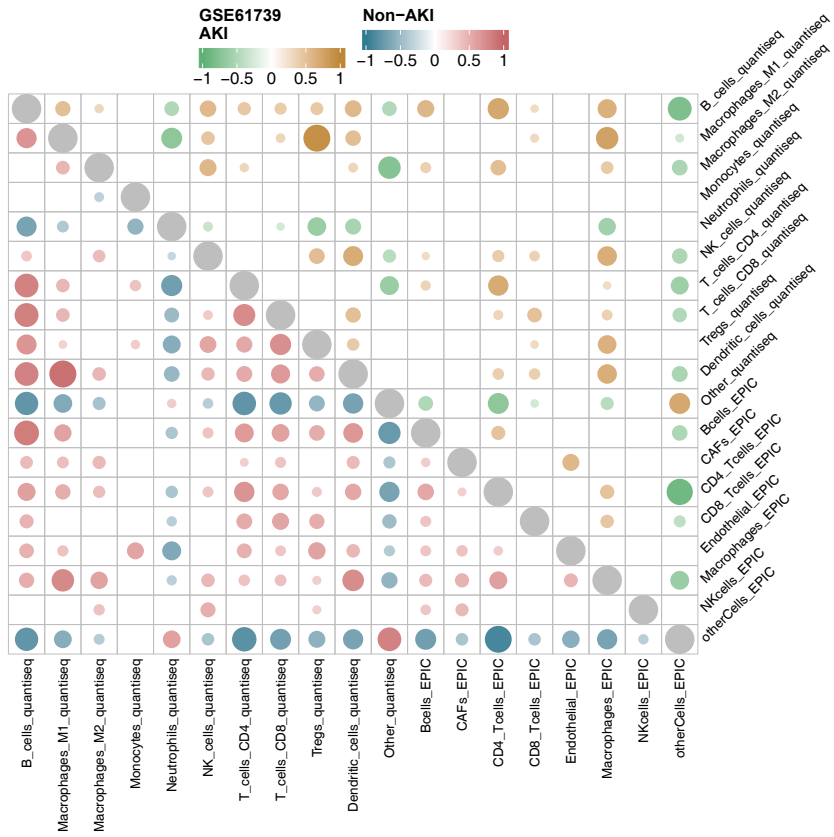

**B**

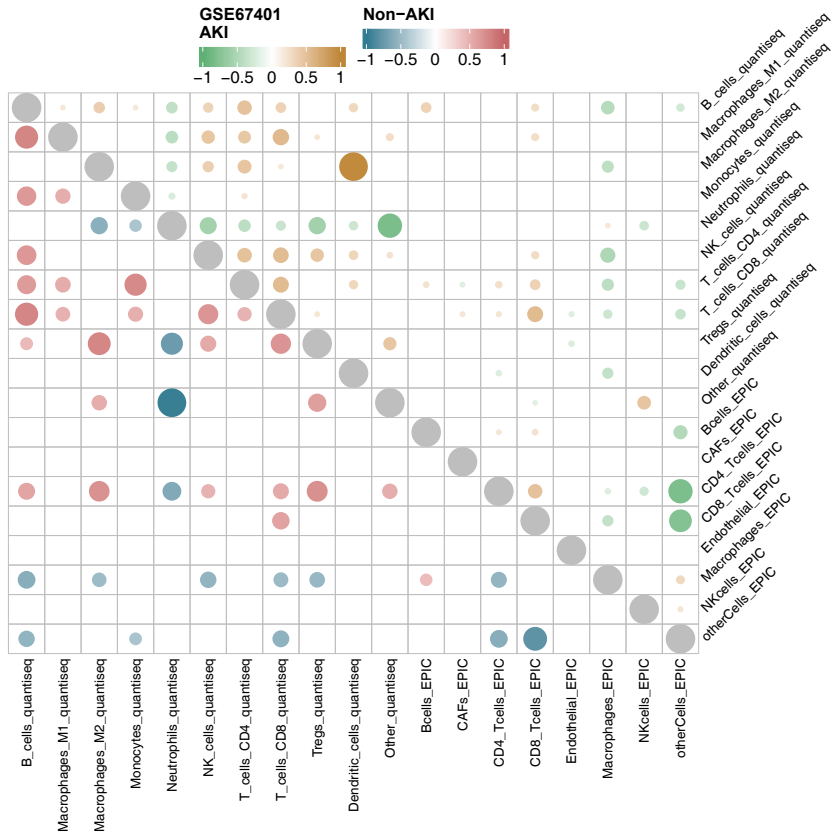

Supplement: Supplementary file 1 — Figure S1 [file JCMM-27-3202-s002.pdf]

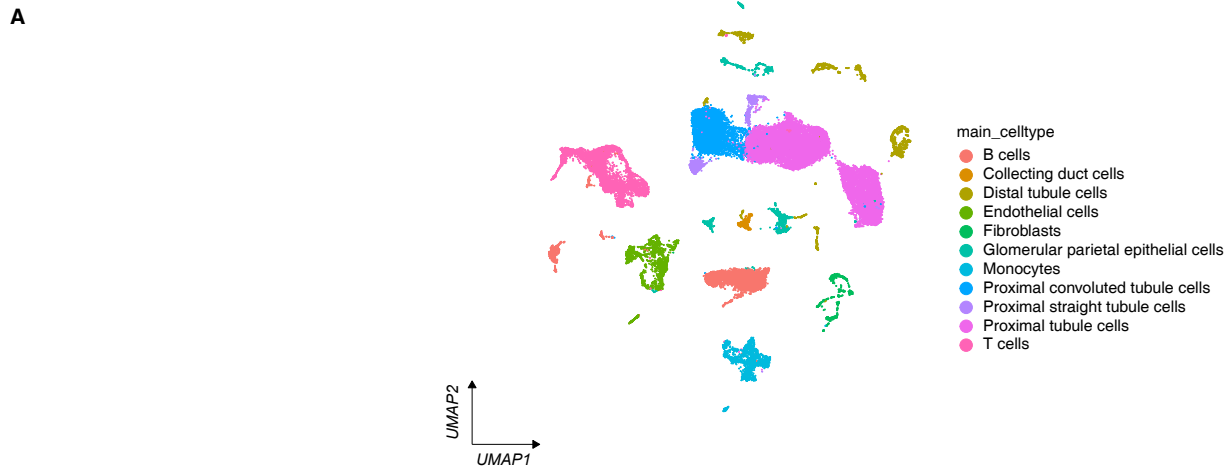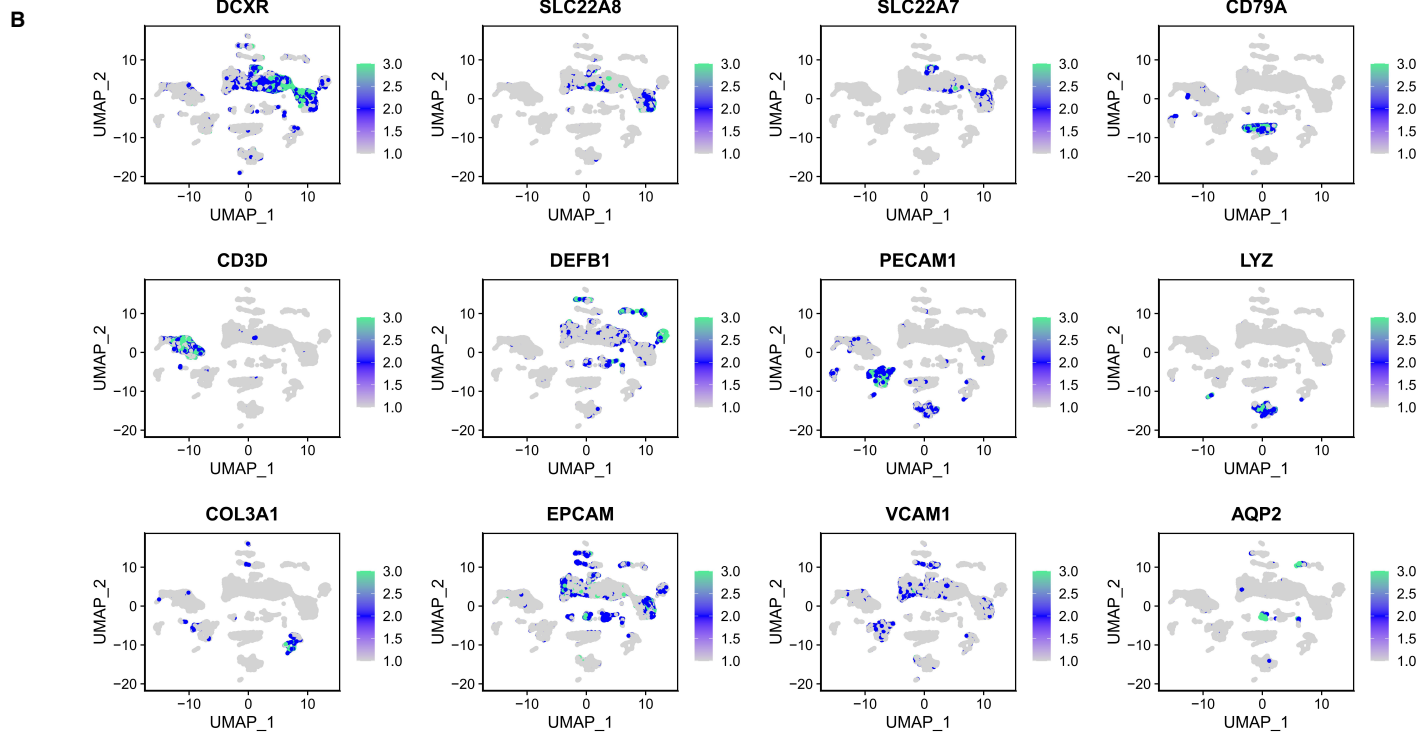

Supplement: Supplementary file 2 — Figure S2 [file JCMM-27-3202-s003.pdf]

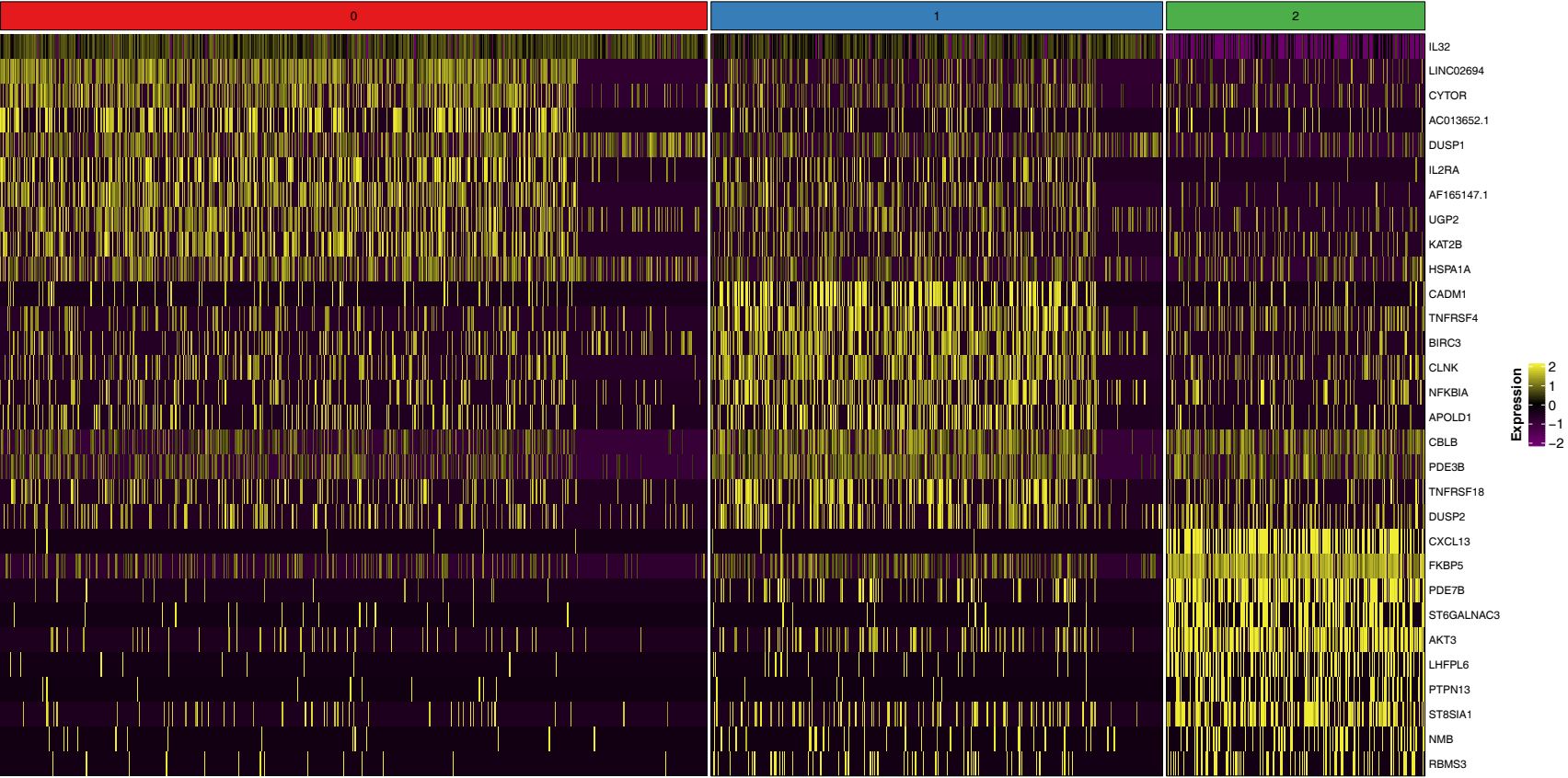

Supplement: Supplementary file 3 — Figure S3 [file JCMM-27-3202-s005.pdf]
