# Supplementary material for: The role of regulatory T cells in the pathogenesis of acute kidney injury
Source: J Cell Mol Med. 2023 Sep 4;27(20):3202–12. doi: 10.1111/jcmm.17771 (PMC10568672; doi:10.1111/jcmm.17771)
Supplement: Supplementary file 4 — Data S1 [file JCMM-27-3202-s004.docx]

**Supplementary Methods**

**Chronic kidney transplatation rejection tissue processing and dissociation**

Two fresh chronic kidney transplatation rejection samples were collected at the Zhujiang Hospital of Southern Medical University. First, fresh samples were taken from the operating room and placed in a solution containing Hank’s bal- anced salt solution (HBSS; WISENT, 311-512-CL) and 1% Antibiotic-Antimycotic (Gibco, 15240062) on ice, which was transported to the laboratory within 20 minutes. Then, 0.5–1 g full-thickness sections of kidney tis- sue were cut lengthwise using surgical scissors. Subsequently, the tissue was washed twice with cold Dulbecco’s phosphate-buffered saline (DPBS; WISENT, 311-425-CL). We placed the tissue on a stainless steel cell filter, crushed the tissue with the plunger of a syringe and washed it with DPBS. We added flushing fluid to a centrifuge tube, and collected the kidney fragments into the centrifuge tube, which was then spun at 350 g for 5 min at 4 °C; we then repeated this step. After discarding the supernatant, we used TrypLETM Express Enzyme (1X, Gibco, 12605010) to further digest the sticky clumps of cells for 5–10 min at 37 °C, then terminated digestion using Dulbecco’s modified eagle medium (DMEM; WISENT, 319-006-CL) containing 10% fatal bovine serun (FBS; Gibco, 10099141). The digested cells were centrifuged at 350 g for 5 min at 4 °C. After discarding the supernatant, the cells were resuspended in 5 ml of DPBS and filtered through a 100 μm cell strainer. Next, we removed red blood cells using 1X RBC lysis buffer (10X diluted to 1X,BioLegend, B250015) for 5 min and centrifuged the cells at 300 g for 5 min at 4 °C. After discarding the supernatant, the cells were suspended in DPBS and centrifuged again. After discarding the supernatant, the cells were resuspended in cold DPBS and passed through a 40 μm cell strainer. Live cells were counted using trypan blue (0.4%, Gibco, 420301) staining. If the cell viability was above 90%, we perform 10x Genomics sample processing.

**10x Genomics sample processing and cDNA library preparation.**

The 10x Genomics Chromium Single Cell 3′ Reagent Kit v3 user guide (https://support.10xgenomics.com/single-cell-gene-expression/index/doc/user-guide-chromium-single-cell-3-reagent-kits-user-guide-v31-chemistry) was used to prepare the single cell suspension. The single cell samples were passed through a 40 μm cell strainer and counted using a haemo-cytometer with trypan blue. Then, the appropriate volume of each sample was diluted to recover 10,000 kidney cells. Subsequently, the single cell suspension, Gel Beads and oils were added to the 10x Genomics single-cell A chip. We checked that there were no errors before running the assay. After droplet generation, samples were transferred into PCR tubes and we performed reverse transcription using a Thermal Cycler (Bio-Rad). After reverse transcription, cDNA was recovered using a recovery agent, provided by 10x Genomics, followed by silane DynaBead clean-up as outlined in the user guide. Before clean-up using SPRIselect beads, we amplified the cDNA for 10 cycles. The cDNA concentration was detected by a Qubit4.0 fluorometer (Invitrogen). The kidney cDNA libraries were prepared referring to the Chromium Single Cell 3′ Reagent Kit v3.1 user guide.

**Single-cell RNA-seq details and preliminary results**

Samples were sequenced by Hiseq Xten (Illumina, San Diego, CA, USA) with the following run parameters: read 1 for 150 cycles, read 2 for 150 cycles, index for 14 cycles. Preliminary sequencing results (bcl files) were converted to FASTQ files with CellRanger(version3.0,https://support.10xgenomics.com/single-cell-gene-expression/software/pipelines/latest/what-is-cell-ranger). We followed the 10x Genomics standard seq protocol by trimming the barcode and unique molecular identifier (UMI) end to 26 bp, and the mRNA end to 98 bp. Then, the FASTQ files were aligned to the human genome reference sequence GRCh38. Subsequently, we applied CellRanger for preliminary data analysis and generated a file that contained a barcode table, a gene table and a gene expression matrix. We carried out preliminary quality control (QC) on the FASTQ files to ensure high quality scRNA-seq data. At the same time, we obtained some basic information about sequencing by a website, such as the number of cells, the median number of detected genes, sequencing saturation and sequencing depth. The strategy of using CellRanger V3.0 and trimming the FASTQ data to 26 bp × 98 bp was used to pre-process the scRNA-seq data and perform downstream analysis.
