# Supplementary material for: The role of regulatory T cells in the pathogenesis of acute kidney injury
Source: J Cell Mol Med. 2023 Sep 4;27(20):3202–12. doi: 10.1111/jcmm.17771 (PMC10568672; doi:10.1111/jcmm.17771)
Supplement: Supplementary file 6 — Table S2 [file JCMM-27-3202-s001.docx]

Table S2. The marker genes for the fifteen cell clusters

| gene | cell type |
| --- | --- |
| DCXR | Proximal tubule cells |
| SLC22A8 | Proximal convoluted tubule cells |
| SLC22A7 | Proximal straight tubule cells |
| EPCAM | Glomerular parietal epithelial cells |
| KRT18 | Glomerular parietal epithelial cells |
| KRT8 | Glomerular parietal epithelial cells |
| CD24 | Glomerular parietal epithelial cells |
| DEFB1 | Distal tubule cells |
| AQP2 | Collecting duct cells |
| CD79A | B cells |
| CD79B | B cells |
| MS4A1 | B cells |
| PECAM1 | Endothelial cells |
| COL3A1 | Fibroblast |
| CD3D | T cells |
| CD2 | T cells |
| CD3E | T cells |
| LYZ | Monocytes |
| CD4 T cells | CD4 |
| CD8 T cells | CD8A |
| Memory T cells | LTB |
| Gama delta T cells | TRDC |
| Treg | CTLA4 |
